# (Not so) Great Expectations: Listening to Foreign-Accented Speech Reduces the Brain’s Anticipatory Processes

**DOI:** 10.3389/fpsyg.2020.02143

**Published:** 2020-08-25

**Authors:** Niels O. Schiller, Bastien P.-A. Boutonnet, Marianne L. S. De Heer Kloots, Marieke Meelen, Bobby Ruijgrok, Lisa L.-S. Cheng

**Affiliations:** ^1^Leiden University Centre for Linguistics, Leiden University, Leiden, Netherlands; ^2^Leiden Institute for Brain and Cognition, Leiden, Netherlands

**Keywords:** prediction, speech perception, sentence comprehension, foreign-accented speech, Dutch, native vs. non-native speech processing

## Abstract

This study examines the effect of foreign-accented speech on the predictive ability of our brain. Listeners actively anticipate upcoming linguistic information in the speech signal so as to facilitate and reduce processing load. However, it is unclear whether or not listeners also do this when they are exposed to speech from non-native speakers. In the present study, we exposed native Dutch listeners to sentences produced by native and non-native speakers while measuring their brain activity using electroencephalography. We found that listeners’ brain activity differed depending on whether they listened to native or non-native speech. However, participants’ overall performance as measured by word recall rate was unaffected. We discussed the results in relation to previous findings as well as the automaticity of anticipation.

## Introduction

Language comprehension involves many tasks such as word recognition including segmentation (where are the boundaries between words?), lexical access (activating word forms and their corresponding meanings stored in memory) and putting word meanings together to understand the message. Our brains are able to fulfill these complex tasks without any problem. How? It has been suggested that one factor contributing to the process of language comprehension and potentially facilitating it is prediction. Our brains may pro-actively predict what is to come next in an utterance and a conversation based on prior knowledge. Some researchers have claimed that the language comprehension system makes use of the predictive power of the brain by predicting upcoming information in the speech stream.

In language processing, but also in a wide array of other cognitive domains (Kok et al., 2012b, 2014; Boutonnet and Lupyan, 2015; Samaha et al., 2016; Vandenbroucke et al., 2016), comprehenders actively anticipate upcoming information thereby (pre-)activating specific features of such linguistic information, ranging from basic acoustic features to high-level conceptual-semantic ones (Federmeier and Kutas, 1999; DeLong et al., 2005; Van Berkum et al., 2005; Obleser et al., 2007; Schiller et al., 2009; Vinck et al., 2011; McGettigan et al., 2012; Van Berkum, 2013; Foucart et al., 2014, 2015; DeLong and Kutas, 2016), in order to facilitate and reduce the processing load (Pickering and Garrod, 2007).

For instance, Schiller et al. (2009) present electrophysiological data from two experiments demonstrating that listeners make predictions for upcoming words using a speech-error detection task. Their data strongly suggest that natural speech is processed semantically even when speech is not task-relevant. Their results further indicate that listeners attempt to predict upcoming words. This process of prediction presumably facilitates comprehension and communication in general. Nevertheless, more recently it has been argued that pre-activation of the phonological form of upcoming linguistic information may not be as stable as suggested by previous research and that prediction may not be a necessary condition for language comprehension (Ito et al., 2017; Nieuwland et al., 2018).

However, the bulk of studies available to date has demonstrated such anticipatory processes in the visual modality in experiments where words are presented one-by-one on a computer screen at a relatively slow and regular pace. Two of the main limitations of such paradigms are that (a) the auditory modality is not involved in such paradigms, and (b) speech communication takes place at a much faster rate (Riding and Vincent, 1980). If anything, visual presentation rate is likely to enhance and entrench predictive mechanisms. Investigations in the auditory modality have so far limited themselves to measuring word anticipation in speech produced and comprehended by native speakers (Foucart et al., 2015) with the exception of studies by Lev-Ari (2015) and Romero-Rivas et al. (2016). However, these studies do not directly investigate anticipatory mechanisms *proper* in the brain.

In today’s society, daily interactions with non-native speakers are becoming more and more frequent, if not the norm. Foreign-accented speech differs from native speech in at least three ways: the presence of non-canonical and unstable phonology (Nissen et al., 2007; Wade et al., 2007; Wester et al., 2007; Hanulíková et al., 2012) followed by unusual prosodic patterns (Gut, 2012) and loose semantic word choice [e.g., the so-called “chocolate pie” vs. “brownie” effect mentioned by Lev-Ari (2015)]. Given that the reason for the brain to predict upcoming information is to facilitate subsequent processing, it is not unreasonable to assume that foreign-accented speech may strongly modulate the nature and involvement of linguistic predictive mechanisms. However, would our brain predict more, less, or just as much when listening to a non-native speaker?

There are at least two ways in which predictive mechanisms in the comprehension of foreign-accented speech might be modulated. The first, and perhaps the most intuitive, is that prediction may increase. Given unreliable and potentially ambiguous input, generating stronger predictions could help a comprehender normalize non-native deviance (Goslin et al., 2012) so as to reduce processing load. The opposite could also be true. Given that foreign-accented speech is more variable (and essentially noisier) than speech produced by native speakers, predictions may fall short of the signal (especially in the case where not enough knowledge is present about the specific ways in which an interlocutor deviates from the native norm) thereby confronting the system with too many prediction errors to be resolved – and consequently increasing processing load.

Two of the most relevant studies on foreign-accent processing available to date point in these two opposing directions. In the study by Lev-Ari (2015), participants, whose eye-movements were being recorded, were presented with arrays of five pictorial items, three of which shared two themes, and two of them shared only one. On each trial, the participants were asked to follow the auditory instructions to click on, e.g., “the witch on a broom,” then on “the man on the magic carpet” and then on “Santa riding a sleigh,” thereby setting up a semantic theme of “imaginary creatures.” Instructions came either from a native or a non-native speaker with a foreign accent. On the next trial, two items would be left on the screen: a *mermaid* and a *ferry*. The *mermaid* shares only the main theme set up by the three items (“imaginary creatures”; i.e., witch, Santa, etc.) and the *ferry* only shares the less dominant one (“means of transportation”; i.e., broom, magic carpet, sleigh). The participants were then instructed to click on the /fεri/, which is interpretable as both “fairy” and “ferry.” The results showed that upon word onset, the participants’ eyes were already fixating more toward the *mermaid* and chose it more often than the (target) picture of the *ferry*, especially when they were instructed by a non-native compared to a native speaker and when they had high as opposed to low working memory load. This suggests that the participants were strongly relying on the context (i.e., imaginary creatures) to anticipate upcoming trials and less on the acoustic speech output of foreign-accented speakers, and this somewhat tricked them into choosing the “wrong” target.

In another relevant study, Romero-Rivas et al. (2016) presented highly constrained high-cloze probability sentences to two groups of native speakers of Spanish. The sentences were produced either by a native or non-native speaker, respectively. Participants’ brain activity was monitored using electroencephalography (EEG). Each of these sentences ended either with a highly probable lexical item (“best completion”), an item semantically related to the best completion or an unrelated item. Earlier research by Kutas and Hillyard (1980, 1984) has shown that such a manipulation results in what has become known as the N400 effect. Processing of all (content) words results in an event-related brain potential (ERP) component with a negative polarity that peaks around 400 ms after word onset and is called the *N400 component*. In their seminal study, Kutas and Hillyard (1980) demonstrated that semantically anomalous words elicit a more negative deflection in the ERP signal than semantically appropriate words occurring in the same position in sentences, i.e., an *N400 effect* (*He spread the warm bread with butter* vs. *He spread the warm bread with socks*). The N400 effect has been associated with lexical and post-lexical processing, e.g., lexical access and semantic integration of words into context (Kutas and Van Petten, 1994; Kutas and Federmeier, 2000; Van Petten and Luka, 2006; for reviews; see also Holcomb, 1993). The N400 effect is currently considered as indicating the difficulty with which a word can be integrated in the utterance context (Leckey and Federmeier, 2019).

By looking at the brain activity following the presentation of the final word, Romero-Rivas et al. (2016) found that while both groups of participants showed increased N400 activity when presented with the semantically related but not expected word, only the group which listened to native speakers showed a difference between the semantically related and unrelated conditions (which yielded further increased N400 activity). In other words, regardless of whether the lexical item was related to the best completion, it required just as much effort to be integrated by the brain. From these results, Romero-Rivas et al. (2016) concluded that anticipation is not affected by foreign accent but that the activation of semantic relationships may not be as “wide” when someone is listening to a non-native speaker – a result consistent with some of the evidence from speech comprehension in adverse conditions (Goslin et al., 2012; Strauß et al., 2013). However, it is important to note that this study does not measure anticipation *per se*, as Romero-Rivas et al. (2016) monitored brain activity starting from the onset of the critical word rather than before the critical word, as is commonly done. At this stage, i.e., the onset of the critical word, brain activity might reflect a mix between the pre-activation and integration of the critical words rather than their pre-activation alone. Finally, because the accent manipulation is applied between-participants in their study, it may be the case that some degree of accommodation to foreign-accented speech has occurred (but see Witteman et al., 2013 who found rapid adaptation to foreign-accented speech even in a within-participants design).

To disentangle these two potential explanations (i.e., whether foreign-accented speech increases or decreases anticipation) and to resolve some of the potential shortcomings from Romero-Rivas et al. (2016) study, we designed an experiment that enables us to measure word anticipation before the presentation of the critical word and manipulates expectedness as well as speaker’s accent within-participants. This design has the advantage of suppressing potential accommodation effects, which might happen in a blocked design, as well as reflecting a more ecologically valid environment where a given person might be communicating with a mix of native and non-native speakers in a short span of time. To do so, we used a classic paradigm including the creation of high-cloze probability sentences while manipulating agreement between the highly expected lexical item and a preceding article (DeLong et al., 2005; Martin et al., 2013) or a preceding adjective (Van Berkum et al., 2005; Otten et al., 2007). More specifically, we adapted the design of a study by Foucart et al. (2015) which consisted of manipulating the grammatical gender of the article (*het* or *de*, in Dutch) preceding the expected or unexpected target while masking the critical noun so as to record brain activity which only pertains to the processing of the article and word anticipation rather than a mix of the two (see section “Materials and Methods” for more detail).

We hypothesized that if anticipation processes take place during speech processing, we should observe modulations of ERP amplitudes depending on whether or not the article matches the expected noun, with increased amplitudes for mismatches. Based on Foucart et al. (2015), we expected to find modulations in an early ERP time window (∼200–300 ms), i.e., a phonological mismatch negativity (PMN), and a later N400 effect. The PMN is a negative-going ERP component shown to be sensitive to phonological properties of words and taken as an indicator of early lexical processing, such as the initial (pre-lexical) phonological processing stage of auditory speech perception (Connolly and Phillips, 1994; Connolly et al., 1995; Schiller et al., 2009). The PMN has been reported, for instance, during the processing of a phonological mismatch in the onset of an expected and an actually heard word. Temporally, the PMN precedes the (auditory) N400 and has been shown to be largely independent of the N400, since it occurred regardless of the semantic appropriateness of the spoken words (Connolly and Phillips, 1994; D’Arcy et al., 2004 for an overview). It is usually identified in the ERP signal as the most negative peak between 150 and 350 ms after stimulus onset.

Furthermore, based on the studies mentioned earlier, we hypothesized that the particular accent (i.e., non-native vs. native) with which a trial was produced should interact with the classic expectedness effect, especially in the earlier time window which we believe is the most likely one to index the pre-activation of stimulus-specific features (Foucart et al., 2015). However, we did not have specific hypotheses regarding the direction of the interaction since the evidence available to date suggests either increased reliance on predictions (Lev-Ari, 2015) or a somewhat more shallow effect of these predictions (Romero-Rivas et al., 2015, 2016), suggesting a potentially less pronounced effect of predictions during non-native accent processing.

Finally, we wanted to assess the impact of native vs. foreign accent on the memory traces generated by word prediction (Foucart et al., 2015). Therefore, we presented participants with a list of words and asked them to indicate whether or not they had heard them in the sentence listening part. The word list consisted of an equal proportion of old expected and unexpected words from the listening task as well as new and neutral (not dependent on a predictive sentential context) ones (see section “Materials” for details). As previously demonstrated (Foucart et al., 2015), expected yet unheard, words seem to form stronger memory traces than unexpected (and unheard) words. We expect that if interactions between expectedness and accent are present at the neural level, an impact on word recall may also be detected.

## Materials and Methods

### Participants

Twenty-four native speakers of Dutch (*M*_*age*_ = 23, *SE* = 0.82) took part in the experiment. All participants were right-handed and had normal or corrected-to-normal vision and reported no neurological nor auditory disorder. Participants were students at Leiden University. The study was carried out in accordance with the recommendations of the local ethics committee at the Faculty of Humanities of Leiden University. All participants gave their written informed consent before taking part in the experiment, in accordance with the Declaration of Helsinki. The data of nine participants were removed due to low signal-to-noise ratio and/or when ≥25% of the trials had to be removed because of non-correctible artifacts. One of those nine participants was also removed due to extremely low accuracy (<40% correct) on the comprehension questions in the listening task.

### Materials

One-hundred-and-twenty highly predictive Dutch sentence contexts were created such that each would generate a strong expectation (≥70% cloze probability – see Supplementary Material) for a particular lexical item. In the present experiment, this lexical item was always a noun of a specific grammatical gender in Dutch (e.g., the neuter noun *boek* ‘book’ preceded by the neuter definite determiner *het* ‘the_*NEU*_,’ or the non-neuter noun *tafel* ‘table’ with the non-neuter definite determiner *de* ‘the_*NON–NEU*_’). Sixty additional sentences were created as filler sentences without strong expectations of any lexical item (see Table 1).

**TABLE 1:** Examples of the sentences (with simple glosses and translation between parentheses for the Control condition) used in the experiment.

Condition	Sentence context	Article + deleted item	Sentence closure
Expected	Mijn gelezen boeken staan op de onderste plank in my read books stand on the lowest shelf in	**de** kast the (book)shelf	op mijn kamer. in my room
Unexpected	Mijn gelezen boeken staan op de onderste plank in my read books stand on the lowest shelf in	**het** bureau the desk	op mijn kamer. in my room
Expected	André is de nieuwe coach van André is the new coach of	**het** team the team	op zijn volleybalclub. in his volleyball club
Unexpected	André is de nieuwe coach van André is the new coach of	**de** groep the group	op zijn volleybalclub. in his volleyball club
Control	Lezen is niet saai, zolang je maar geen saaie boeken leest. reading is not boring, as long you but no boring books read (Reading is not boring as long as you do not read boring books.)	Lezen reading	—
Control	Mijn zoon woont al drie jaar in het buitenland, maar mijn my son lives already 3 years in the foreign land, but my (My son already lives abroad for 3 years, but my dochter studeert en woont nog thuis. daughter studies and lives still at home daughter still studies and lives at home.)	zoon son	—

*Note that for the control sentences, the deletion was not always in a noun phrase or after an article. As explained in the Materials and Methods section (see section “Acoustic Processing”), this was to disrupt the pattern in the experimental sentences.*

### Recordings

All 180 sentence contexts were recorded by a group of four Dutch native speakers (two males, two females) and four non-native Dutch speakers (two males, i.e., one native speaker of German and one native speaker of English [Southern-Irish variety], and two females, i.e., one native speaker of Macedonian, the other of Polish). The decision to include a variety of different accents was so that we would not observe an effect of a particular accent but rather of accented speech in general. Native speakers were asked to pronounce the sentences as naturally as possible and with neutral prosody. Non-native speakers were asked the same but listened to the native speakers’ recordings before recording their own production in order to minimize differences in rate of speech and overall prosody. None of the non-native recordings contained mispronunciations or obvious errors, and the difference between native and non-native recordings was that non-native speakers’ phonetic output was at times non-canonical.

To alleviate potential systematic confounds between the length of expected vs. unexpected sentences (see section “Acoustic Processing”), the assignment of the expectedness conditions was fully randomized for each participant. Each subgroup of sentences was then further divided with one half coming from native speakers and the other from non-native speakers, also randomized for each participant. Thus, each participant was presented only once with a given sentence context, either with an expected or unexpected article and either produced with a native or non-native accent.

**FIGURE 1:**
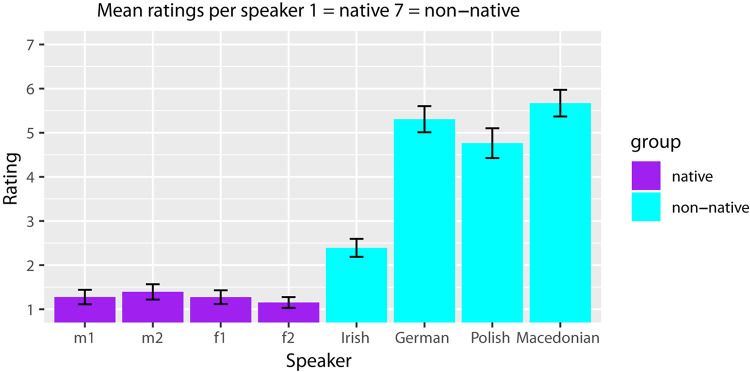
Error bar chart of the mean ratings per speaker.

It has been shown that strong foreign accents have a different effect on listeners’ comprehension than slight accents (e.g., Witteman et al., 2013; Porretta et al., 2017). To obtain an objective measure of the accents of the eight speakers, a separate group of 40 native Dutch participants (*M*_*age*_ = 21.30, *SE* = 0.65) took part in a rating study. These participants completed a Qualtrics survey (Qualtrics, Provo, UT, United States) in which they rated the sentences produced by each speaker on a slider scale (from 1 = “no accent” to 7 = “very strong accent”). All sentences including the fillers were presented auditorily, such that participants were never presented with the same sentence context twice. Participants were paid € 5 upon completion of the task. The native Dutch speakers received a median rating of 1.07 (*M* = 1.26, *SE* = 0.07) and the non-native speakers received a median rating of 4.32 (*M* = 4.35, *SE* = 0.11). These ratings differed significantly from each other, *V* = 0, *p* < 0.001, *d* = 1.11. Figure 1 illustrates the mean ratings obtained for each individual speaker.

To test for differences between the eight speakers, pairwise comparisons using a Wilcoxon signed rank test with Bonferroni correction were calculated and summarized in Table 2.

**TABLE 2:** Means and standard errors of the mean ratings and *p*-values of the Bonferroni-corrected multiple comparisons between the recorded speakers.

			Comparison (*p*-value)						
			
Speaker	Mean	*SE*	m2	f1	f2	Irish	German	Polish	Macedonian
m1	1.28	0.08	0.038	1.00	0.696	<0.001	<0.001	<0.001	<0.001
m2	1.39	0.08	–	0.037	0.001	<0.001	<0.001	<0.001	<0.001
f1	1.27	0.08	–	–	0.095	<0.001	<0.001	<0.001	<0.001
f2	1.15	0.06	–	–	–	<0.001	<0.001	<0.001	<0.001
Irish	2.39	0.10	–	–	–	–	<0.001	<0.001	<0.001
German	5.31	0.15	–	–	–	–	–	<0.001	0.002
Polish	4.76	0.16	–	–	–	–	–	–	<0.001
Macedonian	5.67	0.15	–	–	–	–	–	–	–

Within the native Dutch speaker group, the mean ratings of one male speaker (i.e., “m2”) differed significantly from the three other speakers. Within the foreign-accented speaker group, the mean ratings of all speakers differed significantly from each other. Compared with the native Dutch speaker group, the foreign-accented speaker group was less homogeneous. However, since all participants received equally many sentence contexts from each speaker, any potential advantage from a particular speaker should be equal in all conditions and for all participants.

### Acoustic Processing

Recordings were processed and edited in Praat (Boersma, 2002). To make each condition (expected vs. unexpected) comparable as well as to reduce any bias due to cues such as overall prosody, coarticulation, and so on, we used the same sentence context for both versions of each sentence (either the one recorded for the expected or unexpected condition) up to the word preceding the article and spliced the rest of the sentence from the other condition. Conditions were randomly selected with an equal division between the two conditions. In each of the 120 experimental sentences, the (un)expected noun was completely muted for 500 ms after the article offset and the sentence closure followed this break. The average sentence context length was of 3.05 s (*SE* = 0.03) and that of the article was of 0.16 s (*SE* = 0.001).

To ensure that the purpose of the experiment remained unclear to the participants, we included silences (between 200 and 300 ms long) in the filler sentences at random positions. Finally, to justify the presence of silences to the participants, sentences were band-pass filtered in the range 300–3,000 Hz to make them sound like they were extracted from a telephone conversation. Crucially, for the present study (see the sections “Hypothesis Testing” and “Results”), while sentence-context length and article length were significantly longer in the non-native recordings than in the native recordings (*b* = 0.52, *t* = 3.861, *p* < 0.0002; *b* = 0.03, *t* = 5.37, *p* < 0.0001, respectively), these effects never interacted with the article’s expectedness.

## Procedures

### Listening Task

Participants were seated in a sound-proof testing room and sat approximately one meter from a computer screen and two loud speakers placed on either side of the screen. Participants were told to listen and pay attention to the sentences that were going to be played to them and that they would be asked questions about those sentences during and after the listening phase. Thirty percent of the sentences were randomly followed by a written question on the screen regarding the preceding spoken sentence and requiring a “yes” or “no” response via button presses, the sole purpose of which was to ensure that participants paid attention to the sentences. After a practice of five sentences, participants were auditorily presented with 180 sentences (60 expected sentences [30 native/30 non-native], 60 unexpected sentences [30 native/30 non-native], and 60 fillers [30 native/30 non-natives]). Each sentence was preceded by a black fixation cross on the white screen in front of them for a duration of 1 s after which it turned red for a period of 500 ms to announce to the participant that the sentence was about to start, and then it subsequently turned blue when the sentence started and remained on screen until the sentence ended. A blank screen of 200 ms preceded the next trial. When a question followed the sentence, it appeared on screen following the blank screen and remained visible until participants responded. Another blank screen of 200 ms followed participants’ answers before the next trial started. Participants were invited to take a break every 30 sentences. Their electroencephalogram (EEG) was recorded throughout the listening task of the experiment which lasted 30–40 min.

### Lexical Recall Task

Upon completion of the listening task, participants were presented with a series of words presented one-by-one on the screen. For each word, they were asked to decide whether or not they had heard it in the listening task. These words included the 60 expected nouns, the 60 unexpected nouns (both unheard/muted), and 60 words (heard/presented) from the filler sentences. Word-presentation order was randomized for each participant.

## Data Collection, EEG Pre-Processing and Analyses

The EEG was recorded from 64 Ag/AgCl electrodes placed on the participants’ scalp according to the extended 10–20 convention (American Electroencephalographic Society, 1994) at a rate of 1 kHz in reference to the common mode sense (CMS) and driven right leg (DRL) using a BioSemi (Active Two) system. Data were filtered offline with a high-pass 0.1 Hz filter and a low-pass 20 Hz filter and re-referenced to the common average of all scalp electrodes. Epochs ranging from −100 to 600 ms relative to article onset were extracted from the continuous recording. Epochs with activity exceeding ±75 μV at any electrode site were automatically discarded. The Gratton and Coles algorithm (Gratton et al., 1983) was used to correct for vertical and horizontal eye movement artifacts. Baseline correction was applied in relation to the 100 ms EEG signal of pre-stimulus activity. After these steps, all remaining epochs were averaged by condition and for each participant. Pre-processing steps up to and including eye movement artifact correction were performed in BrainVision Analyzer (Brain Products GmbH) and the remaining steps were performed in the MATLAB environment (v. 2013a – The MathWorks) using a combination of in-house scripts and routines implemented in EEGLAB (v. 13.2.3) and ERPLAB (v. 4.0.3.1).

### Hypothesis Testing

Statistical hypothesis testing on all analyses were performed in the R environment (v. 3.2.3). Linear mixed-effects modeling was performed using the lme4, R package (v. 1.1.10; Bates et al., 2014) and *p*-values from those models were obtained using the Satterthwaite approximation for degrees of freedom implemented in the lmerTest, R package (Tests in Linear Mixed Effects Models [R package lmerTest version 2.0-32], 2014).

#### Behavioral Data

The only behavioral data we analyzed in the present study was the proportion of words recalled by participants. To assess the potential effects of accent and/or expectedness, we used a generalized linear mixed-effect model to predict from the interaction between accent and expectedness by participant whether or not a given word was remembered. We restricted our analysis to the comparison between words which were never heard (due to muting) in the experimental sentences (expected or unexpected) and words which were heard in the control sentences (neutral in terms of expectations).

#### ERP Analyses

Two ERP modulations were identified from grand-averaged data and congruent with previous investigations of word anticipation in the auditory modality (Van Berkum et al., 2005; Foucart et al., 2015). First, we observed an early negative differential in the 120–300 ms time window and, second, a classic N400 effect in the 400–600 ms time window. Both modulations were maximal on electrode Cz and measured over (fronto-)central sites (FC3, FC1, FCz, FC2, FC4, C3, C1, Cz, C2, C4, CP3, CP1, CPz, CP2, CP4). Since the effect was uniformly distributed, and since we were not interested in its spatial distribution, mean ERP amplitudes for the two time periods were measured over the whole central region of interest. For both time windows, mean ERP amplitudes were subjected to a linear mixed-effect model, where mean amplitudes were predicted by the interaction between accent and expectedness, with random slopes by participant.

## Results

### Listening Task (EEG)

#### 120–300 ms Period

**FIGURE 2:**
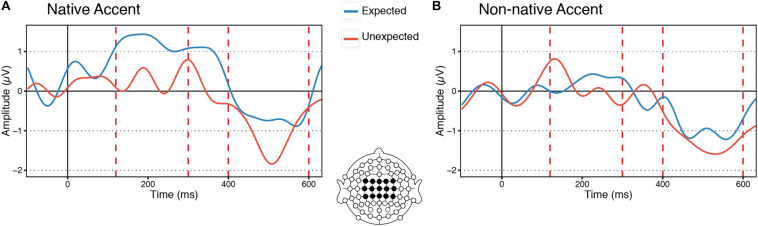
Grand-average waveforms of event-related potentials (ERPs) from the expected and unexpected articles across the central region of interest (FC3, FC1, FCz, FC2, FC4, C3, C1, Cz, C2, C4, CP3, CP1, CPz, CP2, CP4) and across all participants. In blue are ERPs from the expected articles and in red are the ERPs from the unexpected articles. **(A)** ERPs from the articles in sentences presented in a native accent. **(B)** ERPs from the articles in sentences presented in a non-native accent.

The linear mixed-effect model carried out on the first ERP modulation revealed that ERP mean amplitudes could be reliably predicted by an interaction between accent and expectedness (*b* = 0.71, *t* = 3.41, *p* = 0.0007), whereby ERP amplitudes were more negative upon hearing an unexpected article than when hearing an expected article when participants were listening to native speech but this was not the case when they were listening to foreign-accented speech (Figure 2).

#### 400–600 ms Period

In this time window, article expectedness was the only reliable predictor of ERP mean amplitudes (*b* = 0.43, *t* = 2.46, *p* = 0.014), whereby ERP amplitudes were more negative upon hearing an unexpected article compared to an expected one, regardless of the accent (i.e., native vs. non-native) with which the speech was produced (Figure 2).

### Lexical Recall Task

**FIGURE 3:**
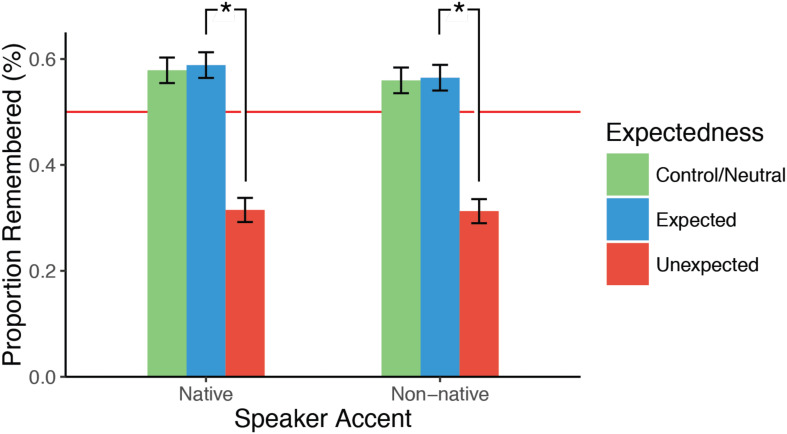
Bar plot showing the proportion of items reported as heard in the sentence listening task. In green are heard words from the control sentences, in blue are unheard but expected words from the experimental sentences, and in red are unheard and unexpected words from the experimental sentences. Error bars depict standard errors of the mean. The red horizontal bar depicts the 50% chance level. *Indicates statistically significant differences (*p* < 0.05).

Results for this task are presented in terms of proportions of words reported as “heard” by the participants resulting in aggregated values between 0 and 1. However, as explained in the “Hypothesis Testing” section above, statistical analyses were performed using logistic regression with the aim to predict a binary label (0 or 1) for each item. The analysis was performed on the unheard expected and unexpected items as well as the heard items from the control sentences. In this task, expectedness reliably predicted whether or not a participant would report a word as heard. In the case where words were actually unheard, participants reported hearing words they had expected significantly more often than words they had not expected (*b* = 1.12, *z* = 7.5, *p* < 0.00001) and, unsurprisingly, participants reported hearing significantly more words that they had actually heard (albeit not strongly expected) compared to words they had not expected (*b* = 1.1, *z* = 7.4, *p* < 0.00001). Importantly, words which were actually heard were recalled just as often as unheard expected words as indicated by the absence of a difference between control and expected sentences (Figure 3).

## Discussion

It is hard to deny that our brain predicts upcoming information. Indeed, prediction and anticipation have been demonstrated in a number of domains ranging across a wide array of cognitive functions such as visual perception, attention and consciousness (Kok et al., 2014; Summerfield and de Lange, 2014) as well as auditory perception (Groppe et al., 2010). Linguistic information has been shown to bias systems as early and as basic as vision (Boutonnet et al., 2013; Lupyan and Ward, 2013; Boutonnet and Lupyan, 2015; Samaha et al., 2016), as well as processes at the interface of these systems (Francken et al., 2014, 2015), and finally, the language system itself (DeLong et al., 2005; Van Berkum et al., 2005; Martin et al., 2013; Van Berkum, 2013; Foucart et al., 2014, 2015).

A key driver of brain predictions is likely to be contextual information. Context influences the content of such predictions (Bar, 2004) as well as their effects on brain activity itself (up-/down-regulation) as is often the case in attention/prediction trade-offs (Kok et al., 2012a, b). While the content of linguistic predictions is known to be influenced by the linguistic context (see “Introduction” section for arguments), we have yet to know whether a given, more general, context can affect how the brain predicts. That is, whether it always predicts or whether its predictions always have the same weight on upcoming information. One of the most common communicative contexts in which people interact nowadays involves people who are communicating in a non-native language. Undeniably, the speech of a non-native speaker is likely to be less accurate in terms of syntax, word choice (e.g., “the chocolate pie” vs. “the brownie” effect; Lev-Ari, 2015), phonological and acoustic realization and so on. If the anticipation mechanism of the brain is set off so that it does not have to fully process upcoming stimuli and it only dedicates the full and costly extent of its processing pipeline when predictions do not match the signal, it is likely that a non-native speaker’s production, which matches canonical predictions less, will affect how our brain predicts.

As reviewed earlier, work by Lev-Ari (2015) demonstrated that participants listening to non-native speech were more likely to rely on contextual information, suggesting that listeners may predict more in such a condition. Conversely, in a study on word integration, Romero-Rivas et al. (2016) showed that integrating the best completion of a sentence (i.e., the most likely lexical candidate given a sentence context) leads to identical brain activity in both native and non-native accent conditions. However, the depth of semantic activation seemed shallower when participants listened to sentences uttered in a foreign accent. While this result cannot speak directly to the issue of anticipation *per se*, it certainly suggests differences in the processing of native versus non-native speech.

To tease apart these preliminary hypotheses, as well as to gain further insights in the anticipatory processes in a manner that is more ecologically valid and at the same time overcoming limitations from between-subject designs, we tested word anticipation in a within-subject design while recording participants’ brain activity using EEG. We expected both early (PMN) and late (N400) ERP components to be modulated by expectedness such that an unexpected article would lead to increased negativities in both time windows (Foucart et al., 2015). We further hypothesized that the expectedness effect should be modulated by the accent with which the sentence was uttered, but we had no strong expectation with regards to its direction due to the mixed results available to date (Lev-Ari, 2015; Romero-Rivas et al., 2015, 2016).

Our results provide support for the fact that the brain does not passively integrate information but, rather, anticipates upcoming words in continuous speech (DeLong et al., 2005; Van Berkum et al., 2005; Otten et al., 2007; Martin et al., 2013; Van Berkum, 2013; Foucart et al., 2014, 2015). Furthermore, we show that context may influence the brain’s predictions as, depending on the reliability of the stimuli, such processes can be up- or down-regulated. Indeed, the simple fact of listening to a non-native speaker reduced the brain’s anticipatory processes as shown by the lack of modulation between expected and unexpected articles in the early ERP time window (PMN). Importantly, reduced anticipation does not mean complete system breakdown as indicated by a similar modulation of the classic N400 (Kutas and Federmeier, 2011; Leckey and Federmeier, 2019) component in both accent conditions. Together with the results from the lexical recall task, which showed that expected items form a more robust memory trace than unexpected items so that participants were just as likely to report a word as “previously heard” when it was expected (but not heard) as compared to when they actually heard the word in either accent conditions, it is clear that successful performance does not depend solely on early anticipation.

The results presented in this study complement earlier findings pointing toward an effect of foreign accent on sentence comprehension (Romero-Rivas et al., 2015) and reinforces the need to measure anticipation *proper* (as done in the present study) since our results are not fully compatible with the supposedly unaffected “anticipation” presented by Romero-Rivas et al. (2016). The interpretation of what processes and mechanisms lead to the early negative deflection are still unclear. However, under an account of the brain as a prediction machine which pre-activates stimulus templates of the sensory input it has predicted (Kok et al., 2014), we believe the early ERP modulation to index feature mismatch at the phonological level given the auditory nature of the experiment as well as the timing, and the topographic distribution of the effect (see also Schiller et al., 2009). This ERP component is often referred to as phonological mismatch negativity (PMN) (Connolly and Phillips, 1994; Hagoort and Brown, 2000; Diaz and Swaab, 2007). This interpretation has also been advanced by Foucart et al. (2015) although they remain quite agnostic as to whether the ERP modulation is an early manifestation of a classic N400 effect or whether it is another component. We believe that our results can help settle that debate, given the fact that while the late ERP modulation – a classic N400 effect – is obtained in both accent conditions, the early modulation is not. This suggests that these two time windows reflect somewhat independent processes: an early feature (phonological) matching mechanism driven by predictions and a late “lexical” recovery/integration process, respectively.

Interestingly, the results from the lexical recall task, showing no interaction between noun expectedness and accent, suggest that the brain is highly flexible and can recover and re-create similar end-of-process effects in behavior. Prediction alone, understood here as the pre-emptive activation of specific stimulus templates, may not be necessary for successful performance in language comprehension – a point recently advocated by Huettig and Mani (2015). That being said, it is important to note that this point does not undermine the fact that prediction is known to take place in several perceptual and cognitive tasks and therefore should not be underestimated. Indeed, further research on linguistic prediction and prediction in the brain in general, preferably including data from more participants, should focus on determining the complex dynamics between predictive and integrative processes to understand the degree of overlap and separation between these processes and their neural and behavioral consequences.

Taken together, our results show that the brain is highly flexible and proactive in language comprehension as well as highly sensitive and responsive to contextual task demands, thereby fine-tuning the influence of higher-level knowledge on lower-level sensory experience, providing strong challenges to models of language comprehension, but also of more general cognition, such as “passive resonance” or other models assuming an almost strictly bottom-up information flow (Biederman, 1987; Serre et al., 2007; Kuperberg et al., 2011; Paczynski and Kuperberg, 2012).

*What are the consequences of the discrepancy between the differences detected in the electrophysiological data and their apparent lack of impact on behavioral performance?* Consider the following point: if brain predictions are the processes by which we think the brain activates specific stimulus templates (Kok et al., 2014), i.e., phonological features in the present case, how can it be that expected *but unheard* words were recalled to the same extent in sentences in which prediction supposedly occurred as well as when it did not? It is important to note that although the early stages of brain activity (potentially related to the activation of phonological features) differed between native and non-native sentences, expectancy effects were obtained in both conditions in the later time window. It must be noted that while the classic N400 effect is often associated with “integration,” given that our stimuli never contained the critical stimuli, the expectancy effect detected on this component cannot come from integration since there is nothing to integrate. Rather, we believe that higher-level conceptual features are likely to have been activated from the highly predictive sentential context at a later stage, yielding a “false” memory trace. Furthermore, we do not exclude the possibility that the activation of higher-level conceptual features can lead to the activation of lower-level perceptual feature as demonstrated in cuing/priming paradigms (Edmiston and Lupyan, 2013, 2017; Boutonnet and Lupyan, 2015; Samaha et al., 2016). Our results are therefore in line with previous reports of such effects on memory by Foucart et al. (2015) and compatible with the evidence showing that degraded speech or phonemes can be restored to the extent that their perceptual experience might not differ from that in optimal conditions (Warren, 1970; Groppe et al., 2010; Sohoglu et al., 2012; Bendixen et al., 2014) and fit very well into theories of perception which allow for higher-level representation to feedback (top-down) information to lower levels. In other words, expecting a specific lexical item, leads to the activation of specific stimulus features across the whole processing stream, which, in case information is absent, resembles that of the actual input (SanMiguel et al., 2013).

## Conclusion

The human brain anticipates upcoming words in on-going conversation. Such anticipation is likely to be supported by predictive mechanisms already identified in various aspects of human cognition and believed to be a key driver of brain function as a whole (Bar, 2003; Friston, 2005; Clark, 2013). However, such predictions may be down-regulated depending on the general context such as stimulus reliability, e.g., whether a sentence is produced by a native or a non-native speaker in the current study. We found that when native speakers of Dutch listened to non-native speakers producing Dutch sentences containing a highly predictable lexical item, their early brain activity did not reveal word anticipation. While brain activity differed depending on whether participants listened to native or non-native accents, their overall performance, measured by word recall, was unaffected, and both accent conditions led to higher recall rates of expected compared to unexpected words, independently of the accent in which the sentences were heard. In other words, listening to a non-native speaker of one’s own native language reduces our brain’s chances to deal with conflicting information only at the levels where the input might be most misaligned with one’s predicted features such as acoustic or phonological features.

## Data Availability Statement

The raw data supporting the conclusions of this article will be made available by the authors, without undue reservation.

## Ethics Statement

The studies involving human participants were reviewed and approved by Ethics Committee of the Faculty of Humanities, Leiden University. The patients/participants provided their written informed consent to participate in this study.

## Author Contributions

NS and LC contributed to the design of this study and the writing including the revision. BB contributed to the design of the study, carried the study out, analyzed the data, and wrote the first draft. MD carried out the study. MM created the stimuli and carried out part of the study. BR carried out the control measurements between the native and non-native speakers. All authors contributed to the article and approved the submitted version.

## Conflict of Interest

The authors declare that the research was conducted in the absence of any commercial or financial relationships that could be construed as a potential conflict of interest.
