# Supplementary material for: (Not so) Great Expectations: Listening to Foreign-Accented Speech Reduces the Brain’s Anticipatory Processes
Source: Front Psychol. 2020 Aug 25;11:2143. doi: 10.3389/fpsyg.2020.02143 (PMC7479827; doi:10.3389/fpsyg.2020.02143)
Supplement: Supplementary file 1 [file Data_Sheet_1.PDF]

Online Cloze Test

One-hundred-and-forty-two sentences were created to generate the strong expectation of a specific noun-phrase. This type of sentence is commonly referred to as a *high-cloze probability sentence*. For example, the context “It was extremely warm in the car during the traffic jam, so Fiona turned on …” will preferably elicit the noun phrase “the air-conditioning” compared to “the radio”, for example. The sentences we created for the current experiment were in Dutch and were designed so that the most expected noun would be of a given grammatical gender, thereby making any other potential candidates of the opposite gender extremely unlikely (cf., rationale and methods in the manuscript). To assess the strength of the expectation and in order to select a controlled subset of sentences which lead to reliable expectations in the main experiment, we carried out an online cloze probability test.

Methods

Participants

Three-hundred-and-fifty native Dutch speakers took part in this test. Participants were recruited via posts on social media and by word-of-mouth. All answers were anonymous. Only the data of the 213 participants who fully completed the task were analysed.

Procedure

We used the Qualtrics web survey application to handle sentence presentation and answer recording from participants. To keep the number of trials per participant within acceptable time constraints, we randomly assigned them to one of four subsets of either

36 or 35 sentences. Each subset was comprised of a random selection of the 142 sentences, averaging to a presentation rate of 53.25 participants per sentence.

Upon landing on the survey page, participants were given clear instructions to carefully read each sentence and to fill the gap indicated by a series of underscores of identical length with the first candidate that came to their mind. The gap always comprised of a noun phrase and the preceding determiner was never given so as not to bias word choice. Once they confirmed that they understood the instructions, each sentence was presented one by one, a box appeared underneath the sentence for participants to type their answer and no time constraints were imposed.

Analysis & Selection

Each participant's answers were automatically analysed by an algorithm programmed to flag answers which were not part of the Dutch language. Flagged answers were then reviewed by two native Dutch speakers, who could either correct the answer in the case of a clear misspelling/typo or leave it uncorrected when no clear match could be found so that it would count as a potential candidate. Importantly, nonword answers were still counted in the analysis to avoid artificially inflating the choice proportion of other candidates.

Cloze probability was obtained by deriving the percentage of times each candidate had been chosen by participants. For example, if 54 out of 54 participants chose "the air conditioning" in the example given above, then that sentence would have 100% cloze probability. Conversely, if only 27 out of 54 participants answered "the air conditioning", then that sentence would have had a 50% cloze probability.

A selection threshold of >= 70% cloze probability was chosen because we wanted sentences which lead to a strong expectation. This threshold resulted in an

average cloze probability of 87.2% (*SD* = 8.7%) largely within bounds of previous similar studies (e.g., Foucart et al., 2015; Martin et al., 2013).
